# Artificial Intelligence in the Detection, Characterization, and Management of Renal Masses: A Narrative Review

**DOI:** 10.7759/cureus.112349

**Published:** 2026-07-09

**Authors:** Hasan F Buali, Tarek Abushloa, Abdulaziz Al Shaibani, Ahmed Bastawisy, Umar S Farouqi, Mohamed Rafie

**Affiliations:** 1 Urology, King Hamad University Hospital, Muharraq, BHR

**Keywords:** artificial intelligence, deep learning, machine learning, partial nephrectomy, radiomics, renal cell carcinoma, renal mass, surgical planning

## Abstract

Kidney tumors are being found more often today because CT and MRI scans are widely used and kidney masses are frequently discovered by chance. Many of these masses are benign. However, most patients still undergo surgery because doctors cannot confirm the diagnosis from imaging alone. Artificial intelligence (AI) refers to computer systems that perform tasks normally requiring human intelligence, including machine learning, in which algorithms learn patterns directly from data, and radiomics, in which quantitative features are extracted from medical images to support diagnosis. These tools offer new ways to improve how kidney masses are detected, characterized, and treated. This narrative review summarizes current evidence on AI applications across the renal mass pathway, covering automated detection, imaging-based characterization, pathological staging, prognosis prediction, and AI-assisted surgical planning, while also outlining current limitations and future research directions. A review of the English-language literature was performed using PubMed and MEDLINE, with studies published between 2018 and 2026 prioritized. AI shows strong performance across all stages of the renal mass pathway. Deep learning models accurately detect and segment renal masses on CT and MRI. Radiomics-based classifiers distinguish benign from malignant lesions and predict tumor subtype without biopsy. Multimodal AI models predict survival with high accuracy and outperform established clinical scoring systems. AI-assisted surgical planning tools support nephron-sparing surgery and predict postoperative kidney function. Wider clinical use requires better prospective validation, more diverse datasets, and improved model transparency.

## Introduction and background

Renal cell carcinoma (RCC) is one of the most common cancers in urology. The American Cancer Society estimated approximately 81,000 new cases and 14,000 deaths in the United States in 2024 [1]. Survival depends heavily on the stage at diagnosis. The five-year survival rate is over 93% for localized disease. It drops to 17% for patients with distant metastasis [1]. Early and accurate diagnosis is therefore very important.

The European Association of Urology (EAU) annually publishes updated evidence-based guidelines for the management of RCC, most recently in 2025 [2]. These guidelines classify renal tumors according to the 2022 World Health Organization (WHO) system and recommend imaging with contrast-enhanced computed tomography (CT), magnetic resonance imaging (MRI), or ultrasound as the standard approach for detection and characterization [2]. Importantly, the guidelines explicitly acknowledge that conventional imaging modalities, including CT, MRI, and contrast-enhanced ultrasound, are unable to reliably distinguish benign entities such as oncocytoma and fat-poor angiomyolipoma from malignant renal neoplasms [2]. This diagnostic gap is the central clinical problem that artificial intelligence (AI)-based tools aim to address.

Imaging technology has changed how kidney tumors are found. CT, MRI, and ultrasound are now routinely used for many different medical conditions. During these scans, kidney masses are often found by chance without the patient having any symptoms. According to the 2025 EAU guidelines, renal masses are incidentally detected via imaging in the majority of cases, including 87% of patients with T1a-stage tumors [2]. Several novel imaging tools have shown promise for improved non-invasive characterization of renal masses [3]. This has led to a significant rise in the number of patients diagnosed with renal masses each year.

Not all incidentally found kidney masses are dangerous. About 20% of small renal masses (SRMs), defined as solid lesions smaller than 4 cm, turn out to be benign after surgery [4]. The EAU guidelines recommend percutaneous renal mass biopsy as a means of avoiding unnecessary intervention for benign lesions, citing a diagnostic yield of 78-97% and specificity of 98-100% for malignancy [2]. Despite this, most patients still undergo surgery because conventional imaging cannot reliably distinguish benign from malignant lesions, and biopsy remains underused in routine practice. AI, encompassing machine learning and radiomics, has been proposed as a solution to improve detection and characterization accuracy on cross-sectional imaging [5]. Unnecessary operations result in preventable loss of kidney function.

Active surveillance is a safe option for many patients with SRMs and is formally endorsed by the EAU guidelines as an alternative to primary surgery for select Bosniak III cystic lesions and small solid masses in elderly or comorbid patients [2,6]. Tumor growth is slow, with a median rate of 0.37 cm per year, and the risk of metastatic progression is low [6]. However, active surveillance remains underused in practice because clinicians cannot confidently confirm that a mass is safe to observe without invasive testing. This is precisely the gap that non-invasive AI-based characterization tools aim to close, offering a path toward more precise, individualized management decisions in line with the broader shift toward precision medicine in oncology.

AI has emerged as a promising tool in urological oncology more broadly [7,8]. It can analyze large amounts of complex imaging data and identify patterns that are invisible to the human eye. Within urology, AI has shown useful results in prostate, bladder, and kidney cancer. Applications range from image segmentation to outcome prediction [9,10]. Despite this promise, current AI tools in this space remain largely investigational. Most published models are retrospective, single-center, and lack the prospective external validation required for routine clinical deployment, a theme that recurs throughout this review.

The renal mass pathway has four main areas where AI can help. The first is automated detection and segmentation of kidney masses on imaging. The second is non-invasive characterization to identify benign versus malignant lesions and predict tumor subtype. The third is pathological staging and survival prediction. The fourth is surgical planning and prediction of postoperative kidney function. This review aims to summarize current applications of AI in the detection, characterization, prognostication, and surgical management of renal masses, while highlighting current limitations and future research directions. The following sections address each of these four domains in turn, followed by a discussion of limitations and future directions.

## Review

Methodology

This is a narrative review rather than a systematic review. A literature search was performed using PubMed and MEDLINE for English-language studies published between January 2018 and June 2026. Search terms included combinations of artificial intelligence, machine learning, deep learning, radiomics, renal mass, renal cell carcinoma, partial nephrectomy, and surgical planning. Studies were considered for inclusion if they addressed AI applications in renal mass detection, characterization, pathological staging, prognosis prediction, or surgical planning in human subjects. Review articles, protocol papers, and studies unrelated to renal imaging were excluded from the core evidence base but were, where relevant, cited separately to provide background or methodological context. Given the substantial heterogeneity across the included studies in imaging modalities, AI model architectures, outcome definitions, and validation strategies, a quantitative meta-analysis was not performed. Findings are instead synthesized narratively, organized thematically according to the clinical stage of the renal mass pathway that each AI application addresses. No formal risk-of-bias or quality assessment tool, such as Quality Assessment of Diagnostic Accuracy Studies - version 2 (QUADAS-2) or Prediction model Risk Of Bias ASsessment Tool (PROBAST), was applied to individual studies, consistent with the scope of a narrative rather than systematic review; this is acknowledged as a limitation in the discussion below.

AI in the automated detection and segmentation of renal masses

Accurate tumor segmentation is the foundation of quantitative imaging analysis. Manual segmentation is slow and varies between readers. Deep learning models can now perform this task automatically and with accuracy close to that of expert radiologists.

Lin et al. developed a 3D U-Net-based deep convolutional neural network for automated segmentation of the kidney, renal tumor, and renal cyst using CT urography data from 441 patients with renal masses [11]. The dataset included 618 kidneys and 309 renal tumors collected from four different CT scanner types, covering a broad spectrum of equipment used in clinical practice. Ground truth annotations were manually created by two experienced radiologists with five and nine years of urological imaging experience. The segmentation model used a two-step cascade architecture. The first step localized the kidney from the full CT image using global low-resolution information. The second step performed fine segmentation from high-resolution localized images. This two-step approach improved kidney segmentation Dice similarity coefficient (DSC) from 0.942 in the first step to 0.978 in the second step [11].

The renal tumor segmentation model achieved a mean DSC of 0.886 in the training set, 0.843 in the validation set, and 0.844 in the test set. Detection performance for renal tumors was excellent, with area under the curve (AUC) values of 0.994, 0.989, and 0.986 in the training, validation, and test sets respectively. For cyst detection, AUC values were 0.870, 0.877, and 0.882 across the same sets [11]. The model was additionally validated in a separate cohort of 35 patients with small renal tumors measuring 1.5 cm or less. In this challenging group, the detection AUC was 0.896, confirming that the model retained useful performance even for very small lesions. Tumor location and morphology influenced segmentation accuracy. Exophytic tumors with greater than 50% external protrusion achieved the highest DSC of 0.910, while endophytic tumors had a lower DSC of 0.747, reflecting the greater difficulty of segmenting lesions that are surrounded by normal parenchyma with similar CT attenuation [11].

Building on single-center segmentation models, a major weakness that must be addressed is limited generalizability across institutions. Cui et al. addressed this directly by designing a multi-center, multi-scanner, multi-sequence MRI study enrolling 988 pathologically confirmed SRM patients across three centers [12]. Independent segmentation networks were developed for each of five MRI sequences. Training was performed on 733 patients from one center using GE equipment. External validation was performed on 81 patients from two other centers, including patients scanned on non-GE equipment. The internal test set achieved median DSC values of 0.769 to 0.855 across the five sequences, with all patients correctly identified in at least one sequence. External validation on non-GE scanners produced median DSC values of 0.523 to 0.785, confirming reasonable generalizability across different manufacturers [12]. This multi-scanner approach is important because it demonstrates that AI segmentation tools can work across the heterogeneous equipment landscape found in real-world hospitals.

Automated segmentation is not just a detection tool. Once a tumor is accurately segmented, radiomic features can be extracted, tumor volume and morphology can be quantified for surgical planning, and serial measurements can be tracked during active surveillance. Automated segmentation is therefore a critical enabler of every downstream AI application discussed in the remainder of this review.

AI and radiomics in renal mass characterization

Having established how AI can detect and segment renal masses, the next clinical question is how AI can help characterize a mass once it has been found. This is where radiomics, the extraction of large numbers of quantitative features from medical images, plays a central role.

What is Radiomics?

Radiomics is the extraction of large numbers of quantitative features from medical images. These features describe the shape, texture, and intensity of a tumor in ways that cannot be seen by the human eye. When combined with machine learning classifiers, radiomic features can be used to predict diagnosis, subtype, and prognosis [13].

Conventional imaging has limited ability to characterize indeterminate kidney masses, as explicitly acknowledged in the 2025 EAU guidelines, which state that CT, MRI, ultrasound, and contrast-enhanced ultrasound are all unable to reliably distinguish benign entities from malignant renal neoplasms [2]. Radiomics offers a way to perform a virtual biopsy using the imaging data that is already available [3]. Technologies including multiparametric MRI, molecular imaging, and AI-assisted radiomics have all shown promise for improving renal mass characterization [3].

Gutierrez Hidalgo et al. reviewed radiomic tools for renal mass characterization and found good accuracy across multiple studies [13]. Radiomics performed well in distinguishing benign from malignant tumors, identifying RCC subtypes, and predicting tumor aggressiveness. However, the studies were mostly small and retrospective. External validation was rarely performed. The authors concluded that while radiomic tools show strong potential, prospective and externally validated studies are needed before clinical adoption [13]. Kuusk et al. similarly reviewed 42 published studies across three categories: benign versus malignant prediction, subtype differentiation, and prediction of nuclear grade and aggressiveness [14]. They found that radiomics was generally superior to expert radiological assessment but emphasized the predominance of retrospective single-center designs as a major quality limitation. They called for prospective validation studies to augment existing management algorithms for SRMs [14]. 

Benign Versus Malignant Classification

Differentiating benign from malignant SRMs is one of the most important problems in renal mass management. Benign tumors such as oncocytoma and angiomyolipoma without visible fat cannot be reliably distinguished from RCC on standard imaging, a limitation explicitly noted in current EAU guidelines [2]. This leads to many patients undergoing surgery that could have been avoided.

Maddalo et al. developed a radiomic model using CT scans from 85 patients who underwent surgery for kidney masses smaller than 4 cm, including 51 RCCs and 34 benign lesions [15]. Benign lesions consisted of 25 oncocytomas, seven lipid-poor angiomyolipomas, and two renal leiomyomas. A total of 108 radiomic features were extracted from portal venous phase CT images using the Pyradiomics library (Computational Imaging and Bioinformatics Lab, Harvard Medical School, US) in 3D-Slicer software (Surgical Planning Laboratory, Brigham and Women’s Hospital, Harvard Medical School, US). The pipeline included redundancy elimination, feature standardization, dataset balancing, and exclusion of non-reproducible features. After feature selection and cross-validation, a 10-feature signature was identified. On unseen test data, the model achieved an AUC of 0.79, accuracy of 0.73, sensitivity of 0.78, and specificity of 0.63 [15]. The variability in these metrics across cross-validation folds was low, with AUC standard deviation of 0.12, confirming model robustness. These results demonstrate that a carefully constructed radiomic pipeline can discriminate RCC from benign renal tumors using routine CT imaging without biopsy, which has direct implications for reducing unnecessary surgery in patients with indeterminate SRMs. 

RCC Subtype Classification and Nuclear Grade Prediction

Predicting RCC subtype and nuclear grade without surgery has direct clinical value. Clear cell RCC (ccRCC) is the most common subtype and accounts for approximately 90% of all RCC cases according to the WHO 2022 classification adopted by the EAU guidelines [2]. It is also the most aggressive subtype. Papillary and chromophobe subtypes behave differently and require different management strategies.

Aymerich et al. built a machine learning model to classify International Society of Urological Pathology (ISUP) nuclear grade in ccRCC using nephrographic-phase CT images from 109 patients with histopathologically confirmed ccRCC [16]. Radiomic features were extracted and assessed for robustness using intraclass correlation coefficients. Feature redundancy was reduced using Pearson correlation and minimum Redundancy Maximum Relevance selection. Three classifiers were trained using eight-fold cross-validation: logistic regression, support vector machine, and random forest. Logistic regression achieved the best performance with an accuracy of 82% and an AUC of 0.86 [16]. SHapley Additive exPlanations (SHAP) analysis identified major axis length, busyness, and large area emphasis as the most influential features. These represent shape and texture information that reflect the greater morphological complexity and heterogeneity of higher-grade tumors. The use of SHAP explainability is clinically important because it allows radiologists to understand which imaging features are driving the model prediction, increasing transparency and clinical trust [16].

Adding pathological whole-slide image features to CT radiomic features further improves grading accuracy. Zheng et al. analyzed 729 ccRCC patients from three independent cohorts: 512 patients from one hospital used for training and internal validation, 175 patients from The Cancer Genome Atlas for external validation, and 42 patients from the Clinical Proteomic Tumor Analysis Consortium for additional external validation [17]. AI algorithms extracted morphological and textural features from both CT images and whole-slide pathology images separately. A radiomics-based signature model using CT images alone achieved an AUC of 0.93 in training and 0.83 to 0.89 across the three external cohorts. A pathomics-based signature model using pathology images alone achieved an AUC of 0.94 in training and 0.88 to 0.90 across external cohorts [17].

The combined multimodal predictive signature model was developed using an XGBoost [18] classifier trained on both CT and pathological features. It achieved AUC values of 0.95, 0.93, and 0.95 across the three independent cohorts, outperforming both single-modality models with statistical significance (P<0.001, DeLong test) [17]. Beyond grading, the multimodal model demonstrated significant prognostic value. Kaplan-Meier survival analysis confirmed that patients classified as high-grade had significantly worse overall survival in both external cohorts (P=0.002 and P=0.01 respectively). Cox proportional hazard analysis showed univariate hazard ratios of 2.542 (95% CI: 1.363-4.741, P<0.001) and multivariate hazard ratios of 1.723 (95% CI: 0.888-3.357, P=0.003), confirming the signature as an independent prognostic factor after adjusting for age, gender, and tumor stage [17]. The C-index values for the two external cohorts were 0.75 and 0.71 respectively.

AI in pathological staging and prognosis prediction

Beyond characterizing a mass at diagnosis, AI has also been applied to predict cancer stage, recurrence, and survival in RCC once a diagnosis of malignancy is established. These tools address a real clinical need, since RCC behavior varies widely between patients and standard staging systems do not always capture this variability accurately.

Yao et al. developed a deep learning model using whole-slide images from 513 ccRCC patients sourced from The Cancer Genome Atlas database [19]. Each pathological slide was cut into multiple 256 x 256 pixel patches. A modified ResNet50 [20] model pretrained on ImageNet [21] was used to extract feature vectors from each patch. The Clustering-constrained Attention Multiple Instance Learning (CLAM) algorithm, which uses an attention mechanism to identify the most diagnostically relevant regions in each slide, was used to train the classification model. Training used a 10-fold Monte Carlo cross-validation strategy to minimize overfitting. External validation was performed on 144 ccRCC cases from an independent commercial biobank, which differed significantly from the training cohort in patient ethnicity and slide preparation techniques [19].

The deep learning model achieved an AUC of 0.875 and an average precision score of 0.809 for predicting pathological stage. The model generated attention heatmaps that visually highlighted the most diagnostically relevant regions in each whole-slide image, with high-attention areas shown in red and low-attention areas in blue. This visualization tool aids pathologists in focusing on the most informative areas within the slide. The CLAM algorithm also generated interpretable attention scores for each tissue patch, providing a degree of explainability that is uncommon in deep learning pathology models [19]. A prognostic risk model was then built using Least Absolute Shrinkage and Selection Operator (LASSO) regression to identify seven key deep learning features. Patients were divided into high-risk and low-risk groups based on the median risk score. Overall survival was significantly worse in the high-risk group (P=0.003). AUC values for predicting one, three and five-year survival were 0.68, 0.69, and 0.69 respectively. External validation confirmed consistent results, with one, three and five-year survival AUC values of 0.60, 0.60, and 0.65. Multivariate Cox regression identified age, stage, and the deep learning risk score as independent prognostic factors [19].

For metastatic disease, Barkan et al. evaluated an AI model to predict three and five-year overall survival in 322 patients with metastatic RCC starting first-line systemic therapy [22]. An ensemble model integrating multiple machine learning classifiers achieved AUC values of 0.786 and 0.771 for three and five-year survival, respectively. It outperformed both the International Metastatic RCC Database Consortium (IMDC) and Memorial Sloan Kettering Cancer Center (MSKCC) prognostic scoring systems, both of which remain the standard risk stratification tools referenced in current EAU guidelines for systemic therapy decisions in metastatic ccRCC [2,22]. Feature importance analysis identified clinical factors that partially aligned with those recognized in traditional Kaplan-Meier and Cox analyses. The authors emphasized that larger prospective multicenter validation studies are needed before this tool can be used to guide treatment decisions in clinical practice.

Multimodal deep learning frameworks that combine CT imaging with clinical variables have shown particular promise. Mahootiha et al. built a 3D convolutional neural network framework incorporating both CT imaging features and systematically selected clinical variables using Spearman and random forest importance scores [23]. The model was trained with a discrete LogisticHazard-based loss function for survival prediction. The best-performing configuration achieved a concordance index of 0.84 and an AUC of 0.80, surpassing previous CT-based RCC prognosis benchmarks in the literature [23]. Integrating clinical context with imaging features reflects how clinicians actually make decisions and produces more meaningful and personalized survival estimates. 

AI in surgical planning and postoperative outcome prediction

Once a renal mass has been characterized and staged, the final clinical challenge addressed by AI in this pathway is surgical planning. Partial nephrectomy is the standard treatment recommended by the EAU guidelines for most localized kidney masses amenable to resection, given its superior preservation of renal function compared with radical nephrectomy [2]. For complex tumors located deep in the kidney or near major vessels, careful planning is essential. AI-based tools now support this planning process through 3D reconstruction, intraoperative guidance, and functional outcome prediction.

Rodriguez Penaranda et al. conducted a systematic review of AI applications in kidney cancer surgical training, identifying 14 eligible studies from 468 screened records [24]. AI applications in the included studies covered surgical workflow analysis, instrument tracking, tissue identification, and 3D model construction. The review found that AI can objectively assess surgical performance by tracking procedural steps and instrument movement. It can provide real-time, personalized feedback during training, which is not possible with traditional surgical education methods. AI-generated 3D models were particularly valued for preoperative planning and intraoperative navigation. The authors identified real-time tracking and image registration as the main remaining technical challenges for AI-guided surgery [24].

Di Dio et al. reported the clinical use of an AI-based Hyper Accuracy Three-Dimensional model for a 54-year-old woman with a 35 x 25 mm anterior upper-pole renal mass requiring robot-assisted partial nephrectomy [25]. The three-dimensional model was built from CT Digital Imaging and Communications in Medicine (DICOM) images using AI combined with computer vision algorithms. Unlike AI alone, which does not yet produce surgical-grade three-dimensional models independently, the combined AI and computer vision approach achieved the accuracy needed for operative decision-making. The model accurately reproduced the spatial relationship between the tumor, the collecting system, and the renal vasculature. This guided the surgical team in selecting a selective tumor-feeding artery clamping strategy, consistent with the EAU guideline recommendation against off-clamp partial nephrectomy except when specifically indicated [2]. The operation was completed in 85 minutes with 14 minutes of warm ischemia. No intraoperative or postoperative complications were recorded. Histopathology confirmed pT1a ccRCC [25].

Predicting kidney function loss after surgery is another important goal, particularly given the EAU guideline emphasis on nephron preservation as the rationale for favoring partial over radical nephrectomy [2]. Amparore et al. developed a machine learning model using data from 556 patients who underwent minimally invasive partial nephrectomy between 2015 and 2023 [26]. The model incorporated 11 patient, tumor, and intraoperative variables including clamping strategy, resection technique, and renorrhaphy type. Nine classifiers were tested. A Random Forest Regressor achieved the highest performance with a prediction accuracy of 89.29%, a mean absolute error of 8.09 mL/min/1.73 m^2^, and a Pearson correlation of r=0.904 with observed three-month postoperative estimated glomerular filtration rate decline (P<10^-42^) [26]. The authors proposed that this model can be used before surgery to simulate functional outcomes based on planned technique, and intraoperatively to guide decisions about clamping strategy and resection margins. 

Summary of key studies

Table 1 summarizes the study design, sample size, AI model architecture, validation approach, and key performance metrics for the primary studies discussed in this review, to facilitate comparison across the different stages of the renal mass pathway.

**Table 1:** Summary of key studies on artificial intelligence applications across the renal mass pathway DSC: Dice similarity coefficient; AUC: area under the receiver operating characteristic curve; ISUP: International Society of Urological Pathology; CNN: convolutional neural network; ML: machine learning; C-index: concordance index; pts: patients.

Study	Application	Sample size	AI model	Validation	Key metric
Lin et al. [11]	Detection/segmentation	441 pts, 618 kidneys	3D U-Net CNN	Internal test + small-tumor cohort	DSC 0.844 (tumor); AUC 0.986 (detection)
Cui et al. [12]	Detection/segmentation	988 pts, 3 centers	Multi-sequence CNN	External, multi-scanner	DSC 0.769-0.855 (internal); 0.523-0.785 (external)
Maddalo et al. [15]	Benign vs. malignant	85 pts	Radiomic ML classifier	Cross-validation	AUC 0.79; accuracy 0.73
Aymerich et al. [16]	ISUP grade prediction	109 pts	Logistic regression + SHAP	8-fold cross-validation	AUC 0.86; accuracy 82%
Zheng et al. [17]	Multimodal grading/prognosis	729 pts, 3 cohorts	XGBoost (CT + pathology)	2 external cohorts	AUC 0.93-0.95; C-index 0.71-0.75
Yao et al. [19]	Staging/prognosis	513 pts + 144 external	CLAM (ResNet50)	External, different ethnicity	AUC 0.875; survival AUC 0.68-0.69
Barkan et al. [22]	Metastatic survival	322 pts	Ensemble ML	Training/hold-out cohort	AUC 0.786 (3-yr); 0.771 (5-yr)
Mahootiha et al. [23]	Multimodal prognosis	Not specified	3D CNN + clinical data	Test cohort	C-index 0.84; AUC 0.80
Amparore et al. [26]	Postop renal function	556 pts	Random Forest Regressor	Test cohort	Accuracy 89.29%; r=0.904

Limitations and future directions

AI tools for renal mass management show consistent performance gains in research studies. However, several barriers must be addressed before these tools can be used in everyday clinical practice.

The most common problem is that most studies are retrospective and single-center. Models are trained and tested on data from one hospital. When applied to patients from a different institution, performance often drops. This limits how widely these tools can be used [13,14]. The Lin et al. segmentation study, despite using four different CT scanner types, was still conducted at a single center, and the authors acknowledged that only the corticomedullary phase was used, which may not capture all diagnostically relevant information available from other CT phases [11]. The MRI model used in Cui et al. showed that performance on non-GE scanners was lower than on GE equipment, confirming that cross-manufacturer generalizability remains an unsolved challenge [12]. A systematic review of AI in cancer imaging confirmed that real-world clinical validation is lacking in most published studies [27].

Bias in AI models is a serious concern. Bias can enter at any stage of development, from data collection to model deployment [28]. Yao et al. specifically acknowledged that its training data may not represent the broader global patient population due to ethnic and geographic underrepresentation [19]. The Zheng et al. study was conducted entirely in Chinese patients at hospitals in Wuhan, raising questions about generalizability to other populations [17]. When certain patient groups are underrepresented in training data, the model performs worse for those groups. Solutions include using larger and more diverse datasets, applying statistical bias correction methods, and requiring transparent reporting of model performance across subgroups [28].

Many AI models are difficult to interpret. Clinicians cannot always understand why a model made a specific prediction. This reduces trust in the tool and makes it harder to identify errors. The use of SHAP values in the Aymerich et al. and Zheng et al. studies [16,17] and attention heatmaps in the Yao et al. study [19] are early steps toward interpretable AI. However, these methods are not yet standard across the field. Limited explainability, alongside data bias and lack of prospective trials, remains one of the main obstacles to AI adoption in clinical medicine [27].

Beyond the technical limitations of the AI models themselves, this review has its own methodological limitations that should be acknowledged. As a narrative rather than a systematic review, the selection of included studies was not governed by predefined, exhaustive eligibility criteria, and a formal Preferred Reporting Items for Systematic reviews and Meta-Analyses (PRISMA)-style screening process was not undertaken. No formal risk-of-bias or quality appraisal tool, such as QUADAS-2 for diagnostic accuracy studies or PROBAST for prediction models, was applied to the included studies. This approach is appropriate for a narrative synthesis intended to provide clinicians with a broad, accessible overview of a rapidly evolving field, but it carries an inherent risk of selection and publication bias, and readers should interpret the reported performance metrics with this in mind.

Future work should focus on prospective multicenter trials with clear clinical endpoints, ideally designed in alignment with existing clinical guideline frameworks such as those published by the EAU, to ensure that AI tools are evaluated against clinically meaningful comparators [2]. Standardized reporting of AI model design, training data characteristics, and validation methodology is also needed. Federated learning, which allows models to be trained across multiple hospitals without sharing patient data, offers a practical path to building more diverse and generalizable AI tools while respecting patient privacy.

## Conclusions

AI is changing how kidney masses are detected, characterized, staged, and treated across the full clinical pathway. Deep learning models can automatically detect and segment renal masses on CT and MRI with high accuracy, offering a reliable foundation for downstream analysis. Radiomics-based classifiers show strong potential to distinguish benign from malignant SRMs and to predict tumor grade without the need for biopsy. Multimodal models that combine imaging with pathological or clinical data demonstrate meaningful prognostic value and, in several studies, outperform established clinical risk scoring systems for both localized and metastatic disease. AI-assisted three-dimensional surgical planning tools support nephron-sparing decision-making, and machine learning models show promise in predicting postoperative kidney function. Before these tools can be adopted into routine clinical use, they require prospective multicenter validation, more diverse training datasets, improved model explainability, and clearly defined regulatory pathways. With continued progress in these areas, artificial intelligence has the potential to reduce unnecessary surgery, improve diagnostic accuracy, and support more personalized care for patients with renal masses.
